# Do Customers Pay Attention to Motivations and Switching Costs When They Terminate Their Relationships?

**DOI:** 10.3389/fpsyg.2020.00798

**Published:** 2020-04-29

**Authors:** Pan Huifeng, Hong-Youl Ha

**Affiliations:** ^1^Department of Economics, Shanghai University of Political Science and Law, Shanghai, China; ^2^Department of International Trade, Dongguk University, Seoul, South Korea

**Keywords:** relationship termination, moderating effects, relationship avoidance, switching costs, motivations

## Abstract

Research on some key boundary conditions and outcomes of consumers’ relationship termination in the online environment is scare. We examine how four categories (e.g., upkeep, time, benefits, and personal loss) of avoiding relationships affect customers’ relationship termination. We also consider both the motivation (hedonic vs. utilitarian) and switching costs when customers evaluate whether to exit from or stay in a relationship. Results show that time plays a significant role in customers’ relationship termination, but there appears to be an increase or decrease in customers’ relationship termination associated with the role of two moderators. More specifically, upkeep plays a significant role in affecting relationship termination for consumers motivated by hedonic interests (as opposed to those motivated by utilitarian interests). Meanwhile, personal loss plays a role in affecting relationship termination for utilitarian consumers (and not hedonic). Furthermore, we found that high switching costs facilitate a relationship termination if time and personal loss are involved. The findings indicate that the effect of high switching costs on customer loyalty is limited. We also found that when consumers consider time category, they are likely to have a greater intent to terminate a relationship regardless of the level of switching costs.

## Introduction

Firms with improved digital experiences are more able to leverage their customer relationship management (CRM) profile to attain superior customer satisfaction outcomes (Srinivasan and Moorman, 2005). However, many managers at marketing and business conferences express concern about their performance and have been asking questions such as “Why is our CRM system failing?” and “Why do consumers not want to have a relationship with us?” Similarly, researchers also question the effectiveness of customer defection in a digital retail context (Srinivasan and Moorman, 2005; Ha and Janda, 2011). A fruitful way to further understand this issue may be to a glean further understanding of what consumers really want to avoid in their existing relationships (Fournier, 1998). In this case, relationship termination is related to the experience with or perception of a firm.

Numerous studies in psychology and marketing have addressed consumer behavior toward switching, fading, and terminating relationships. As highlighted in Table 1, researchers investigating the relationship fading or termination focus mainly on the impact of cognitive and emotional variables such as expectations, quality perceptions, and negative feelings. Their focus on relationship fading and termination is on attitude movement in positive and negative directions (Evanschitzky et al., 2020). However, little is known about the actual psychological traits that drive relationship termination. Specifically, why and in what way consumers tend to terminate long-term relationships in online settings is a question that has not received enough empirical attention (Ashley et al., 2011). We use Noble and Phillips (2004) concept of relationship avoidance (i.e., upkeep, time, benefits, and personal loss) to further investigate the above research questions. Although their study provides insights into consumer types of avoidance in the traditional market context, their findings also help to further elaborate on relationship termination in the travel firm context. More specifically, testing the study of Noble and Phillips (2004) is adequate in the online travel firm context, where sharing communication about customer needs, interests, and concerns is needed.

**TABLE 1:** Overview of marketing literature highlighting the relationship fading and termination.

Authors	Context	Design	Key moderating variable	Keyinternal (mental) variable
Fajer and Schouten, 1995	B2C (product-related)	Conceptual	Level of loyalty	Unmet expectations for brand performance, changing Consumer needs/liking criteria
Fournier, 1998	B2C (brand)	Qualitative	–	–
Gronhaug et al., 1999	B2C	Three life-history cases	–	Perceptions of company, sales representatives
Mittal and Lassar, 1998	B2C (health/car repair)	Qualitative	Level of satisfaction/interpersonal interaction	Technical/functional quality perception
Hocutt, 1998	B2C (services)	Conceptual	Commitment	Reactance to high exit barriers, dissatisfaction
Roos, 1999	B2C (product)	Switching pass analysis	Irrevocable/revocable switching paths	Negative feelings (anger, distress, shame, stress, and dissatisfaction)
Tuominen and Kettunen, 2003	B2C (airline services)	Qualitative	Light/medium user	Overall service evaluations
Åkerlund, 2004	B2C (financial service)	Qualitative/quantitative	Economic climate, stock market conditions	Expectation, decreasing, commitment, dissatisfaction quality perception
Noble and Phillips, 2004	B2C	In-depth interview	–	Maintenance, time, benefit loss, personal loss
Monga and Houston, 2006	B2C (product)	Experimental	Prior attitude, performance ambiguity	Expectations change
Hollmann et al., 2015	B2B	Qualitative	–	Relationship external events
Leonidou et al., 2018	B2B relationships	Empirical	–	Negative feelings (anger, loss of trust, and disappointment)
Evanschitzky et al., 2020	B2C (fading stages)	Qualitative	–	Negative surprise, dissatisfaction, frustration, anger, distrust
This study	B2C (travel)	Empirical	Level of shopping motivations/switching costs	Upkeep, time, benefit, personal loss

There are several theoretical approaches to better understand relationship termination. Motivation theory may offer a useful framework when multiple needs remain unmet, resulting in frustration (Hanna and Wozniak, 2001). The use of social-exchange theories capable of explaining relationship termination in a digital consumer behavior setting has been somewhat limited. The notions of perceived effort and perceived loss may be useful in understanding the mechanism of relationship termination (Noble and Phillips, 2004) and privacy in online settings may be a critical trade-off in relationship performance (Winer, 2001; Ashley et al., 2011).

Although these theoretical frameworks can be adapted to the context of digital relationship termination, some unique aspects of this study present several new challenges. First, most digital firms have implemented tracking mechanisms that monitor whether a customer responds to CRM systems. Such systems may be useful for a more complete understanding of customer actions, but CRM systems cannot fully predict the feelings and behavior of consumers who terminate relationships. Furthermore, Noble and Phillips (2004) and Ha (2015, 2017) had mainly focused on key factors of relationship avoidance (or termination) from the cross-sectional to longitudinal perspectives. That is, research that has addressed relationship termination on the internet is limited, suggesting that this research area is still in its infancy. By a better understanding of relationship termination and how they relate to intent to terminate a relationship, we aim to fill in this gap and contribute meaningfully to the extant literature.

To synthesize the research in this domain, particularly, we look at the role of motivation (hedonic vs. utilitarian) and switching costs when customers evaluate their intent to leave or remain in a relationship. In particular, the efficacy of alternative moderating mechanisms is conditional on relational exchange factors (Poppo et al., 2016). The switching costs and shopping motivation associated with moderating mechanisms reflect relational termination impacts, especially in emerging markets where customer-oriented market supporting systems are underdeveloped (Ha and Lee, 2012). Furthermore, customer motivation and relevant costs related to relationship termination is still in its infancy in the tourism literature (Ha, 2017), indicating that a better understanding of two boundary conditions is mandatory. These moderators can play an important role in terminating or managing the current relationship with a website; thus, a more complete understanding of these factors can help marketing organizations in online settings further improve their marketing efforts and bolster the probability of maintaining a relationship with customers.

The remainder of this paper begins by addressing the research background, establishing research hypotheses, describing the research methodology, and testing the proposed hypotheses. This is followed by a discussion of the key findings, a summary of limitations, and an outline of future research directions.

## Conceptual Development

### Background of Termination Behaviors

Although most researchers and practitioners recognize the value of relationship marketing (RM), the effectiveness of RM can depend on several factors such as prior customer–brand experiences (Fournier, 1998), risk avoidance (Gu et al., 2017), relationship avoidance (Noble and Phillips, 2004; Grégoire et al., 2009; Ashley et al., 2011; Ha and Lee, 2012; Ha, 2015, 2017), relationship fading (Evanschitzky et al., 2020), or anti-consumption behavior (Lee et al., 2009). This section starts with a summary of prior experiences that are well documented in the relationship marketing literature and subsequently presents a review of research related to digital relationship termination behavior.

A prior negative experience in an online setting may encourage the intent to end a relationship (Ha and Lee, 2012). Furthermore, Fournier (1998) found that the main reason of relationship ending is closely related to negative experiences with a particular brand. Therefore, a negative prior experience can become a critical element in the decision to enter or continue in a relationship if the negative performance fails to meet the accepted level of customer expectations (Zeithaml et al., 1993).

The connection between relationship termination and prior experience is that digital users will tend to patronize websites where they can easily assess performance, and in turn, they will end those that are difficult to judge. Thus, relationship termination is a result of perceived experience differences between positive and negative experience levels. That is, the bigger the perceived negative difference, the less likely a website will be selected.

Although the key assumption of relationship marketing is that consumers prefer to form a relationship to obtain desired benefits, many obstacles can hinder relationships with a website. One of these pertains to unenticing benefits, a situation where the customer feels that the benefits offered by the retailer are not sufficient enough to warrant the time and effort involved in maintaining the relationship (Noble and Phillips, 2004). This is also directly related to the anticipated benefits that address the relationship obstacles that result from failing to recognize purported benefits or having concerns about whether sustaining a relationship is worthwhile (Ashley et al., 2011).

Both anti-consumption and anti-choice behaviors may be useful for a complete understanding of relationship termination with certain products or brands. Take the instance of a consumer visiting a website (or downloading a mobile application) that offers attractive benefits; however, he/she is disappointed because that which was offered has either sold out or gone away after a promotional period. As with the expectation–performance linkage of services proposed by Zeithaml et al. (1993), undesirable behavior usually occurs when unmet expectations lead to negative first-hand consumption experiences (Lee et al., 2009). This is linked to a prevention focus for avoiding risks associated with future negative consequences (Briley and Wyer, 2002).

Furthermore, the concept of marketing avoidance is beneficial in explaining consumers’ desire to shield themselves from marketing promotions and protect privacy (Hann et al., 2008). As Fournier (1998) noted, avoidance behavior to protect consumer privacy is often caused by firms’ marketing activities that impose inconvenience or other negative outcomes for consumers (Hann et al., 2008). Given that these activities are perceived to be undesirable by consumers, they may motivate consumers to avoid having a relationship with the firm (Ha and Lee, 2012). For this research stream, the approach avoidance framework has demonstrated the negative implications of undesirability for achievement-related outcomes (Roney et al., 1995; Elliot and Sheldon, 1997), resulting in the reinforcement of avoidance behavior.

Table 2 distinguishes this study from the only two other studies that have mainly focused on relationship avoidance behavior from the traditional market (Noble and Phillips, 2004) and online market (Ha, 2017). In general, this study is the first to focus on the role of motivation and switching cost in decisions to end a relationship. In particular, we provide theoretical and managerial contributions for extending the literature.

**TABLE 2:** Studies that focus on four themes of relationship avoidance.

	Noble and Phillips, 2004	Ha, 2017	This study
Consideration of an interaction between four themes and relationship exit	×	×	v
Consideration of relationship exit	×	×	v
Consideration of moderating variables	×	×	v
Key objective	Identify drawbacks to the consumers, which is a critical endeavor for understanding why consumers avoid relationship building programs.	Investigate relational dynamics between four themes and relationship avoidance over time.	Exam how relationship avoidance influences customer intent to leave and how moderators involve in the proposed relationships.
Key findings	Four themes of relationship hindrance (e.g., upkeep, time, benefit, and Personal loss themes).	Upkeep theme is insignificant, whereas time and personal loss themes play a crucial role in terminating the relationship.	Upkeep losses are not significant, whereas time and benefit losses influence customers’ intent to leave. Both utilitarian and low switching cost play an important role in bridging the proposed relationships.
Key implications for future research	Investigate long-term relationships, which might be difficult to exit.	Identify key moderating factors that can change in the relationship between four themes and relational exit.	Cultural difference and point of purchase should receive more attention.

### Relationship Termination

Relationship termination is a more advanced construct than are ones centered on the theme of anti-relationship. The former focuses on the actual negative attitude toward an entity (e.g., firm, online marketer), whereas the latter focuses on critical obstacles that negatively affect relationship building. Relationship termination is thus defined here as “a consumer’s attitudinal desire to exit the relationship with a particular website.” This definition is consistent with Park (2010) finding that, when people experience or face negative consequences, they may respond with avoidance-motivated goals that are consistent with their underlying motivations. These avoidance-motivated goals should be either directly or indirectly linked to forming a negative attitude toward a particular object (Gable, 2006; Impett et al., 2010). It is also postulated that a gap between consumer expectations and these reasons will facilitate the formation of a negative attitude.

Even though relationship termination may still be an unfamiliar construct in the marketing literature, research on the topic has been garnering attention in psychology, organizational behavior, and marketing. Consumers will likely avoid uncertainty in some types of relationships and are likely to evaluate such relationships in terms of perceived loss and perceived effort (Noble and Phillips, 2004). Consumers tend to make a trade-off between perceived loss and effort. Perceived loss is a critical construct because it can contribute to consumer dissatisfaction with a firm (for instance, when using websites or mobile applications) and over time lower the probability of forgiveness (McCullough et al., 2003) and raise the likelihood of relationship endings (Grégoire et al., 2009). It thus seems fruitful to further understand conditions that lead consumers to seek relationship avoidance and how that affects the intent to end a relationship.

### Research Hypotheses

**FIGURE 1:**
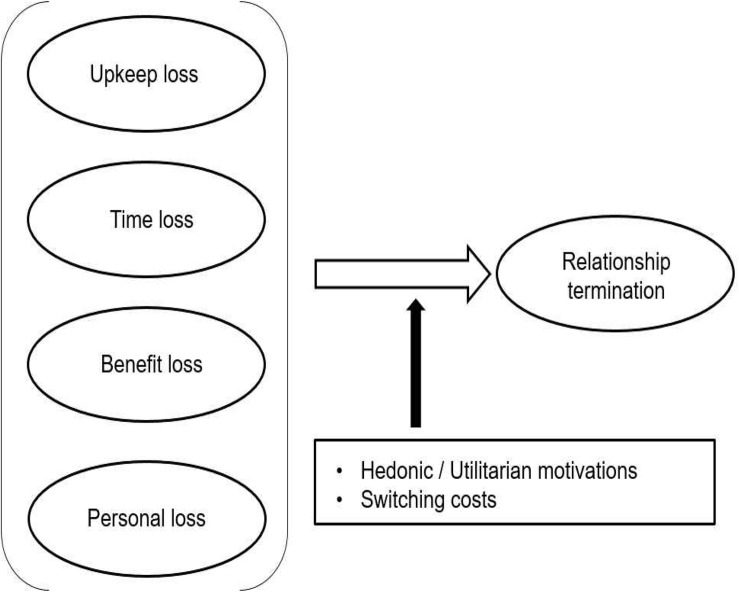
Proposed model.

The proposed research model is derived from the literature on consumer behavior in online shopping and relationship contexts (Figure 1). Relationship termination may arise from a variety of factors that have been well articulated by Noble and Phillips (2004). According to Noble and Phillips (2004), upkeep reflects the annoying tasks that consumers feel they have to engage in, to maintain their relationship with a provider. Time represents the time required to initiate or maintain a relationship with a website (or mobile application). Benefit represents a consumer’s belief that some problem exists with the benefits offered through relational programs. Finally, personal loss represents consumer perception of loss associated with privacy and/or social issues.

Relationship termination is closely related to a consumer’s belief that there is some relationship loss in a current relationship or potential relationship with a particular website (or mobile application). Attachment theory draws from personal relationship research to suggest that when environmental conditions change in an unexpected and/or threatening manner, a series of behaviors can potentially be triggered in which people often reestablish their behavioral patterns (Hazan and Shaver, 1992). The concept of relationship loss plays a central role in research streams such as the personal relationship-based view, relationship marketing, neural science, behavioral economics, and psychological dynamics (Schoenbachler and Gordon, 2002; Tom et al., 2007; Adams et al., 2008).

As the relational obstacles increase, relationship termination generated by time losses, benefit losses, and psychological losses becomes more tacit. For example, customers are reluctant to waste time on a website to maintain a relationship. However, most website practitioners often misunderstand the importance of time convenience. This suggests that they must improve time convenience to keep customers as customers are afraid to lose their precious time (McKeown, 2002). An economic benefit is another example. If relationship maintenance becomes more difficult, users may consider abandoning the relationship (Evanschitzky et al., 2020). In sum, these three loss categories that constitute relationship termination essentially should reflect the important role of personal loss in the relational process. Thus, we propose the following three hypotheses:

H1. Time loss is positively associated with relationship termination
H2. Benefit loss is positively associated with relationship termination
H3. Personal loss is positively associated with relationship termination

However, arguably, these four loss categories may not equally influence customer’s intent to leave, because these types are dynamic over time. For example, a recent study empirically illustrates that the upkeep loss category does not exist for customer relationship termination over time (Ha, 2017). Because most digital users are well aware of this issue, they are more likely to ignore upkeep category compared with the initial stages of online shopping or tend to form indifferent attitudes toward system processes. This trend leads to relationship maintenance crisis. Customers may misunderstand the website strategies by believing that the maintenance announcement is ineffective. In the study, we propose the method to limit the effect of the upkeep loss category on relationship termination. Thus, we propose the following hypotheses:

H4. Upkeep loss is not positively associated with relationship termination.

### Moderating Role of Hedonic/Utilitarian Motivations

Web-usage theory (Cotte et al., 2006) provides the underpinning theoretical perspective on the role of hedonic/utilitarian motivations in affecting the linkage between relationship loss and relationship endings. According to this framework, individuals form a motivational foundation for their continued participation in or interactions with a particular website, thus suggesting that their hedonic/utilitarian motivations determine their future behavior (Pöyry et al., 2013). Furthermore, the nature of relational participation will affect motivation for decisions aimed toward avoiding post-behavioral negative consequences (Simonson, 1992). Utilitarian motivations will thus focus on the efficiency of achieving specific goals and minimizing inefficiencies.

Since efficiency is related to utilitarian motivations, customers are likely to be more tolerant of other features of the online (or mobile) experience as long as efficiency is maintained. This allows utilitarian customers to minimize their potential losses. From an efficiency perspective, upkeep and benefit losses are principally utilitarian, which customers often use for almost purely functional purposes (Rychalski and Hudson, 2017). For example, if a customer perceives potential functional losses in a relationship, the customer is likely to terminate the relationship. Thus, we propose the following two hypotheses:

H5. Customer motivations positively moderate the effect of upkeep loss on relationship termination.
H6. Customer motivations positively moderate the effect of benefit loss on relationship termination.

Meanwhile, hedonic motivations will lead to a greater emphasis on the flow experience (Novak et al., 2003) as opposed to utilitarian motivations that would emphasize efficiency (Cotte et al., 2006). The flow experience characterized by exploratory browsing, which can involve variety seeking and risk taking is central to a hedonic motivation, will allow the customer to be more inclined to immerse himself/herself in this flow. However, hedonic customers are more sensitive to a relationship, especially when they have dissatisfied experience. They will not return to a website owing to their bad experience (Bougie et al., 2003). In this study, customers motivated by negative hedonic motivations such as time loss and personal loss traits are likely to terminate a relationship if they have dissatisfied experience or do not feel real entertainment. As such, these two categories will have a greater effect on intent to terminate a relationship (Novak et al., 2000; Voss et al., 2003). Thus, we hypothesize the following:

H7. Customer motivations positively moderate the effect of time loss on relationship termination.
H8. Customer motivations positively moderate the effect of personal loss on relationship termination.

These hypotheses, H5–H8, mean that hedonic and utilitarian motivations positively moderate the effect of upkeep loss (other three loss categories such as time, benefit, and personal loss). Because this study identifies two groups, namely hedonic and utilitarian motivations, it is possible that a customer may have hedonic and utilitarian motivations both, either hedonic or utilitarian motivations only. To reconcile these issues, this study especially compares the differences of the moderating effects between the two groups.

### Moderating Role of Switching Cost

Switching cost is defined here as the perception of the degree to which additional economic, psychological and emotional costs are required to terminate the current relationship and secure an alternative (Sharma and Patterson, 2000; Jones et al., 2002). Prior research suggests that the moderating effect of switching costs is useful for a better understanding of the customer relationship process (Lee et al., 2001; Jones et al., 2002). When switching costs are particularly high, customers would find the thought of switching particularly painful. Hence, customers will learn how to take the necessary economic and behavioral steps to maintain their current relationships (Yang and Peterson, 2004).

Conversely, when switching costs are perceived to be low, customers will be less inclined to invest the time and effort (e.g., upkeep and benefit) to maintain the relationship. Thus, upkeep, time, and benefit loss categories accelerate the dissolution because switching costs have a potential ability to end the relationship (Halinen and Tähtinen, 2002). Furthermore, a switch or transaction termination will conditionally occur if a customer perceives any anxiety and fear (Ongena and Smith, 2001). This is also conditional if switching costs are low as customers may be reluctant to defect the current relationship with other service providers owing to high switching costs. Thus, four categories will be more strongly related to intent to terminate a relationship. Accordingly, we propose the following hypothesis:

H9. Switching costs positively moderate the effect of upkeep on intent to leave a relationship.
H10. Switching costs positively moderate the effect of time on intent to leave a relationship.
H11. Switching costs positively moderate the effect of benefit on intent to leave a relationship.
H12. Switching costs positively moderate the effect of personal loss on intent to leave a relationship.

## Research Methods

### Research Setting

The research setting for data collection involved digital travel markets operated by large-scale travel companies in South Korea. Unlike smaller travel markets in South Korea, most travel markets that use digital platforms are designed, communicated, and are managed very systematically. For such markets, however, the switching costs are relatively lower than for single-owned small markets (Park and Ha, 2012). Therefore, CRM among travel markets is a fundamental priority, because South Korean travel markets have become extremely competitive due to the presence of global markets such as expedia.com, tripadvisor.com, hotels.com, and trivago.com. As such, this research setting is particularly desirable for examining the nature of relationship avoidance.

### Data Collection

We collected data from an online market-research firm (tillionpanel.com) to gain access to commercial market users. The research firm contacted digital market users across South Korea (within its 428 panelists who met the study’s criteria), and 300 users agreed to participate. All participants were registered on digital commercial websites (via either a computer or a mobile device) and had a minimum of 6-month browsing experience prior to data collection, indicating that they were eligible for inclusion in the study.

We employed a short-term data-collection procedure to minimize any possible response bias. Meanwhile, to ensure that respondents answer all questions (and to eliminate missing responses), we embedded a survey platform that did not allow respondents to move on to the next question if they did not respond. Thus, data were collected from these experienced users for the third week of February 2016. Respondents aged 21–59 years were asked to participate; approximately 40% were males and 60% were female. Of the respondents, 31% had a monthly income below US$2,000. The income of the remaining 69% broke down as follows: US$2,000–3,000 (25%), US$3,000–4,000 (15.7%), US$4,000–$5,000 (11%), and >US$5,000 (17.3%). Finally, approximately 67.2% of respondents used mobile platforms when they shopped.

We also checked the data for differences between the travel groups. First, we assessed the non-response bias by analyzing the differences between the respondents (*n* = 300) and non-respondents (*n* = 128) for key descriptive variables. No significant differences were found between the two groups in terms of age (*p* = 0.14) or gender (*p* = 0.19), indicating no non-response bias in the data. This study maintained disaggregated individual-level data from these results.

### Measures

The independent variables modeled to influence customers’ intent to leave are the four relationship avoidance categories: upkeep, time, benefits, and personal loss. In addition, hedonic/utilitarian motivations and switching costs were included as moderating variables. All constructs were obtained on a five-point Likert scale with range “1 = *strongly disagree*” to “5 = *strongly agree.*”

Noble and Phillips (2004) did not originally develop the four categories of relationship avoidance, whereas Ha (2015) developed full scales of relationship avoidance based on Noble and Phillips (2004) original avoidance categories. However, several sub-dimensions have been modified, because customer behavior and IT development have rapidly changed. As shown in Appendix, the items of four categories used in this study were measured using Ha (2015) new measurement scales. More specifically, upkeep was measured using four items of two sub-dimensions such as account maintenance and unnecessary requirements, which have been widely used in the retail and digital marketing literature (Noble and Phillips, 2004; Ha and Janda, 2011; Ha, 2015). Time was measured using four items with the two sub-dimensions: tiring initiation and discordance of/information search. Tiring initiation is directly linked to the initiation, as proposed by Noble and Phillips (2004), but discordance of/information search is new because the original two categories of accumulation and travel were mainly focused on traditional retailing contexts (offline markets). Benefit was measured using five items related to three sub-dimensions (preconditions, poor benefits, and relative suspicion). These categories are very similar to Noble and Phillips (2004) avoidance of purchase requirements (hollow, unenticing, and unknown). Personal-loss was measured using four items with the two sub-dimensions of personal information exposure and technical anxiety. Personal information exposure is widely accepted in marketing, IT, and psychological studies, whereas technical anxiety is new and its relevance has been pointed out in recent literature (see Pavlou, 2003; Lee et al., 2011).

Intent to leave was originally developed from the business-to-business (B2B) relationship literature and has been operationalized as the propensity to terminate the primary relationship partner (Ping, 1993). The notion of customers’ intent to leave in this study was very similar to the B2B intent to leave; hence, the original scales were adapted, and the final version used three items.

We included two variables to investigate the moderating effects in the relationship between relationship avoidance and its outcomes. Hedonic/utilitarian motivations were measured using four items of the utilitarian/hedonic motivations adapted from Babin et al. (1994); Cardoso and Pinto (2010), and Yim et al. (2014). We identified two groups (hedonic and utilitarian motivations) by calculating the mean from four items (*M* = 3.84; hedonic motivation = 153 vs. utilitarian motivation = 147). Switching costs were measured using three items adapted from Jones et al. (2000). Similarly, two groups (low switching costs vs. high switching costs) were identified by calculating the mean (*M* = 3.11; low switching costs = 153 vs. high switching costs = 147).

### Measure Validation

We used the confirmatory factor analysis (CFA) using AMOS 21.0 to assess convergent validity, discriminant validity, and reliability of the constructs. As shown in Appendix, all construct measures showed good psychometric properties. For example, all Cronbach’s alphas were acceptable, indicating that they had high reliability.

We tested measurement validity using an estimated CFA model that included all constructs. The overall model fit was significant, χ^2^(303) = 666.463 (*p* < 0.001), and other indices showed good fit (CFI = 0.939, TLI = 0.909, and RMSEA = 0.067). Based on these statistics, the first step was to evaluate convergent validity by inspecting item loadings. All items loadings were in the range 0.656–0.880, and thus exceeded 0.6, which is the suggested threshold value (Fornell and Larcker, 1981). Next, we calculated composite reliability (CR) using the procedure suggested by Fornell and Larcker (1981). All CRs were above the threshold (CR > 0.7), and the average variance extracted (AVE) also exceeded the threshold value of 0.5, indicating that the measurement model had a good internal consistency. Finally, we assessed the discriminant validity as suggested by Fornell and Larcker (1981). As shown in Table 3, the smallest AVE exceeded the highest squared correlation in the correlation matrix, providing evidence for discriminant validity.

**TABLE 3:** Measurement information andcorrelation matrix.

Construct	Mean (SD)	1	2	3	4	5	6	7
1. Upkeep loss	2.80 (1.21)	**0.570**	–	–	–	–	–	–
2. Time loss	2.79 (1.12)	0.687	**0.527**	–	–	–	–	–
3. Benefit loss	3.13 (1.12)	0.284	0.330	**0.533**	–	–	–	–
4. Personal loss	3.57 (1.15)	0.422	0.436	0.635	**0.594**	–	–	–
5. Relationship termination	2.30 (1.02)	0.280	0.416	0.318	0.121	**0.604**	–	–
6. Hedonic/utilitarian motivations	3.84 (0.71)	-0.080	-0.043	-0.047	0.039	0.031	**0.508**	–
7. Switching costs	3.11 (0.91)	0.001	-0.121	0.105	0.084	0.030	0.099	**0.606**

*Bold numbers on the diagonal show the AVE.*

### Control Variable

Gender was used as a control variable to reduce the alternative hypotheses in the proposed relationships. The control variable was measured by the gender difference (male vs. female) to investigate the difference in relationship termination. Gender is measured in B2C studies.

### Common Method Bias

As upkeep and time losses are highly correlated when measured in the same survey, we checked common method bias in surveys. We performed Harman’s one-factor to test this correlation. In so doing, we input all self-report variables into a principal component factor analysis using varimax rotation to clarify the relationship between factors. Our analysis revealed a seven-factor structure in which each factor was less than 50% of the covariation. We concluded that no general factor was apparent.

### Data Analysis

We first used structural equation modeling (SEM) to test path analysis for observed variables without moderators. We used SEM without moderators to select the best model compared with other alternative models. As most structural modeling is nested, the priority is to compare their research models with the alternative model (Lin et al., 2017). Moreover, previous studies rarely used SEM to test interaction hypotheses (e.g., Bell et al., 2005; Eisingerich and Bell, 2008). Alternative mixed tests are performed using other analytic approaches (Tomarken and Waller, 2005). We applied PROCESS because our proposed model represents two conditional processes (Hayes, 2013). This study particularly focuses on the conditional effect. It estimates how much two cases that differ by one unit on an independent variable are estimated to differ on a dependent variable when a moderator equals some specific value (Hayes, 2012, p. 5). Recently, studies and academic conferences recommended that PROCESS is a useful approach to test conditional effects and the index of moderated mediation (Hayes et al., 2017).

## Results

### The Structural Model and Hypotheses

We analyzed the proposed model without moderating effects (Model 1). In particular, we additionally tested an alternative model without the direct effects of the four categories for the outcome variable (Model 2). That is, Model 2 was a hierarchical model of relationship avoidance with four categories. Model 1’s overall statistics indicated that the model was a good fit for the data [χ^2^(166) = 440.155, χ^2^/*df* = 2.651; CFI = 0.913; TLI = 0.898; RMSEA = 0.077]. Similarly, Model 2 indicates an acceptable fit of the model to the data [χ^2^(165) = 475.711, χ^2^/*df* = 2.883; CFI = 0.858; TLI = 0.821; RMSEA = 0.081]. As shown in Table 4, we investigated Models 1 and 2 using completely standardized path coefficients.

**TABLE 4:** Results of estimated path coefficients.

	Standardized coefficient	Hypothesis	Support
**Model 1: Proposed model** **Control variable**			
Sex	0.061 (ns)	–	No
Time loss ? Relationship termination	0.340^∗∗^	H1	Yes
Benefit loss ? Relationship termination	0.325^∗∗^	H2	Yes
Personal loss ? Relationship termination	0.177^∗∗^	H3	Yes
Upkeep loss ? Relationship termination	0.111 (ns)	H4	No

Model 2: Alternative model			

Relationship loss ? Relationship termination	0.409^∗∗^	H1	Yes
Model comparison	AIC	BIC	
Model 1	1,028.155	1,191.122	
Model 2	1,235.461	1,422.598	
	?AIC = 207.306	?BIC = 231.476	

*^∗^*p* < 0.05; ^∗∗^*p* < 0.01.*

The proposed model (Model 1) uncovers some interesting results. The first was to check the effect of the control variable, revealing that gender was insignificant. This finding indicates that gender differences are homogeneous. Next, we considered the link between four-loss categories and relationship termination with the limited effect of upkeep in H4 (H1–H4). As expected, three categories of relationship termination were positively significant (H1: time, ß = 0.340, *p* < 0.01; H2: benefit, ß = 0.325, *p* < 0.01; H3: personal loss, ß = 0.177, *p* < 0.01). However, the upkeep loss category on relationship termination was insignificant (H4: ß = 0.111, *p* > 0.05). These effects are particularly notable, when consumers have potential relationship termination. These findings were also valuable because the effects of upkeep category in the results of H4 were very limited. This highlights the need to reconsider the relevance of upkeep in the context of online consumer behavior.

Meanwhile, our findings were supported by the results of Model 2. However, Model 2 had a weakness in addressing H4 because Model 2 could not give a detailed account of the differences for each effect of the four loss categories. This led to a comparison of the accuracy of these two models, through which both AIC and BIC were adopted to select the best model. As shown in Table 3, ?AIC and ?BIC were both well above the recommended threshold value of 10 (Burnham and Anderson, 2002). Thus, the proposed model (Model 1) exhibited a suitably better fit for the data and provided additional support for our hypotheses about the differing effects of these four loss categories.

### Moderating Effects and Hypotheses

To test the moderating effects (i.e., H5–H8 and H9–H12), we conducted PROCESS analysis as proposed by Hayes (2013), where bootstrap was used to test a moderation effect. This approach is beneficial to test hypotheses about the mechanisms behind causal effects, which describes and explores the conditional mechanisms by which causality operates.

**FIGURE 2:**
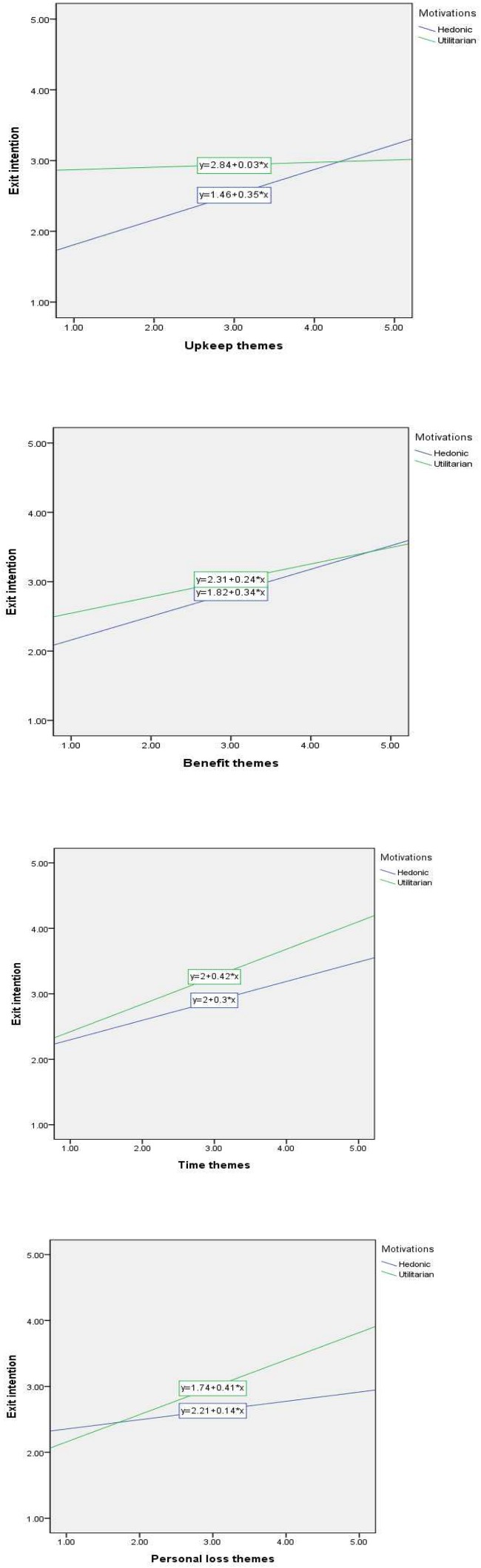
**(A)** The moderating role of hedonic/utilitarianmotivations (H5: Upkeep). **(B)** The moderating role of hedonic/utilitarian motivations (H6: Benefit). **(C)** The moderating role of hedonic/utilitarian motivations (H7: Time). **(D)** The moderating role of hedonic/utilitarian motivations (H8: Personal loss).

H5, H6, H7, and H8 predicted the positive moderating effect of customer motivations between four categories and relationship termination. As shown in Table 5, most moderating effects of customer motivations on the four categories–relationship termination link were statistically supported; however, the effects of customer motivations were split by the different roles of hedonic/utilitarian motivations. More specifically, similarities and differences exist between hedonic and utilitarian motivations. Although the upkeep–relationship termination link (H5: ß = 0.354, *p* < 0.05) is only positively improved by the moderating role of hedonic motivations, the personal loss–relationship termination link (H8: ß = 0.410, *p* < 0.01) is only positively improved by the moderating role of utilitarian motivations. As shown in Figures 2A,D, there appears to be an increase in customers’ relationship termination associated with the high level of hedonic/utilitarian motivations. Both the benefit–relationship termination (H6: hedonic, ß = 0.340, *p* < 0.05 vs. utilitarian, ß = 0.236) and time–customers’ relationship termination (H7: hedonic, ß = 0.296, *p* < 0.05 vs. utilitarian, ß = 0.420, *p* < 0.01) links are positively moderated by two motivations. However, as shown in Figure 2B, when a customer has a more utilitarian focus in the time category context, then relationship termination increases.

**TABLE 5:** Conditional effect of four categories on relationshiptermination at values of the moderators (by PROCESS = 1).

**Path**	**Motivations**		**Significance**	**Original interaction**
	**Hedonic**	**Utilitarian**		
H5: Upkeep loss ? Relationship termination	0.354^∗^	0.034 (ns)	Partially supported	0.320^∗^
H6: Benefit loss ? Relationship termination	0.340^∗^	0.236^∗^	Fully supported	0.103 (ns)
H7: Time loss ? Relationship termination	0.296^∗^	0.420^∗∗^	Fully supported	0.123 (ns)
H8: Personal loss ? Relationship termination	0.140 (ns)	0.410^∗∗^	Partially supported	0.279^∗^
	Switching costs		Significance	Original interaction
	Low	High		
H9: Upkeep ? Relationship termination	0.447^∗∗^	0.082 (ns)	Partially supported	0.530^∗∗^
H10: Time ? Relationship termination	0.365^∗∗^	0.373^∗∗^	Fully supported	0.012 (ns)
H11: Benefit ? Relationship termination	0.402^∗∗^	0.083 (ns)	Partially supported	0.319^∗^
H12: Personal loss ? Relationship termination	0.207 (ns)	0.359^∗∗^	Partially supported	0.151 (ns)

*^∗^*p* < 0.05; ^∗∗^*p* < 0.01.*

Hypotheses, H9–H12, posit that switching costs positively moderate the relationship between four categories and relationship termination. Apart from the three insignificant effects in two groups, most five paths were significant. That is, H10, the time–relationship termination link, was supported in both low and high switching costs, whereas the other three hypotheses, H9, H11, and H12, were partially supported.

**FIGURE 3:**
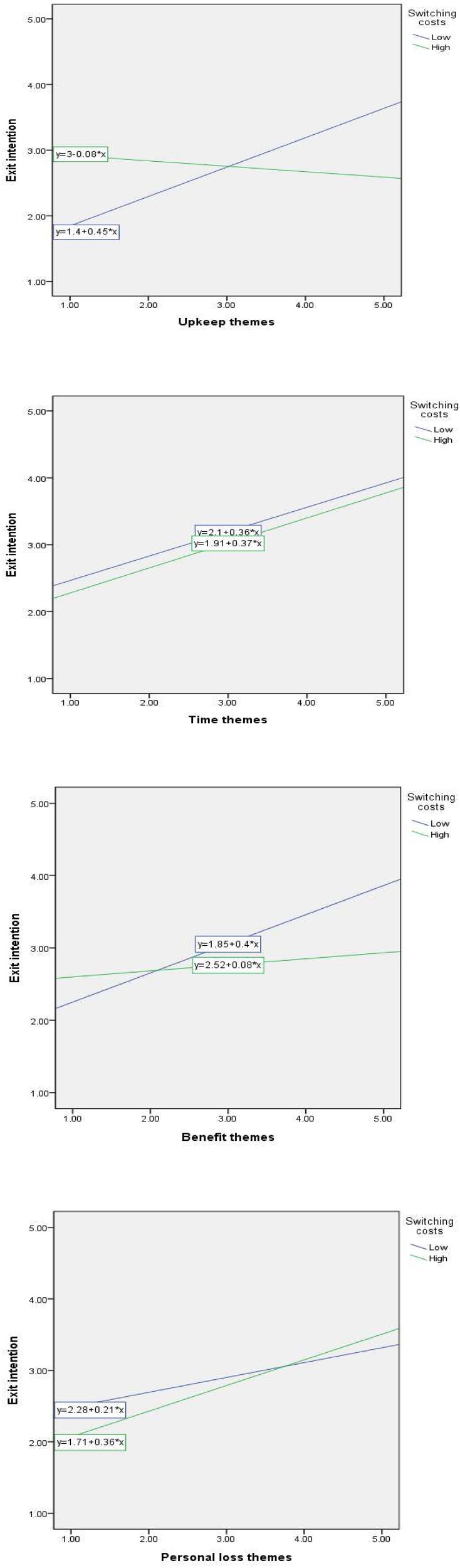
**(A)** The moderating role of switching costs (H3: Upkeep). **(B)** The moderating role of switching costs (H3: Time). **(C)** The moderating role of switching costs (H3: Benefit). **(D)** The moderating role of switching costs (H3: Personal loss).

There were differences that could increase or decrease the proposed relationship rather than the effects of hedonic/utilitarian motivations. Although both the upkeep–relationship termination (H9: ß = 0.447, *p* < 0.01) and the benefit–relationship termination links (H11: ß = 0.402, *p* < 0.01) are only positively improved by the moderating role of low switching costs, the personal loss–relationship termination link (H12: ß = 0.359, *p* < 0.01) is only positively improved by the moderating role of high switching costs (see Figures 3A–D).

Interestingly, a similarity exists between motivations and switching costs. Time was involved in relationship termination, regardless of the role of either moderator. Furthermore, when a customer was more focused on the high switching cost rather than low switching costs, there appeared to be a decrease in the customers’ relationship termination; however, high switching costs do not indicate customer loyalty, because both time and personal loss facilitate customers’ relationship termination.

## Discussion

### Summary of Findings

Our empirical findings from digital consumers establish that relationship loss can significantly affect intent to leave a relationship. These findings are consistent with the literature in marketing and psychology (Fournier, 1998; Noble and Phillips, 2004; Ha and Lee, 2012), but differences also exist in the literature (Ha, 2017). For example, Ha’s study (2017) demonstrated that the overall effect of upkeep loss decreases, whereas our findings show that hedonic motivations and low switching costs positively moderate upkeep on customers’ relationship termination. Specifically, these two moderators in this study increase the relationship between upkeep loss and customers’ relationship termination.

Furthermore, the effect of upkeep, benefit and personal-loss on relationship termination also depends on the level of switching. Specifically, we found that high switching costs facilitate relationship termination relatively if time and personal loss are involved. The findings indicate that the effect of high switching costs on customer loyalty is limited. We also found that when consumers consider time loss category, they are likely to have greater intent to terminate relatively regardless of the level of switching costs. The following section provides an overview of both the theoretical and practical implications of these findings.

### Theoretical Implications

Results of this study have implications for literature related to CRM, which thus far mainly focuses on either a negative relationship (Noble and Phillips, 2004), or non-relationship behavior (Ha and Janda, 2011; Ha, 2015). Conceptually, our study makes a contribution by looking at an important outcome (relationship termination) and exploring how the four loss categories (upkeep, time, benefit, and personal loss) influence this outcome. We also look at the moderating role of two relevant factors (utilitarian vs. hedonic motivations, and switching cost) and how these factors affect the relationship between relationship loss and relationship termination.

We found empirical evidence that relationship termination increases when both hedonic motivations and low switching costs are involved in upkeep. The significant increase between upkeep and relationship termination implies that the consideration of hedonic motivations and low switching costs in deciding whether to leave leads to active moderating effects and thus may facilitate a strong relationship termination. These findings contribute to extant motivation research, which largely focuses on consumer switching behavior (Chiu et al., 2005).

For theory, the findings bolster the effect of high switching costs by highlighting aspects that prior studies have scarcely considered. Most researchers agree that high switching costs are a useful approach not only for explaining the key effect of CRM on customer loyalty but also for understanding how high switching costs protect customers from competitors. Given that time and personal-loss represent a positive relationship termination, by showing that high switching costs increase the relationship between these two categories and relationship termination, we offer evidence for this overestimated effect of switching costs.

Finally, we advance the better understanding of switching costs in the customer-firm value link. We extend this study by establishing the moderating roles of a relational exit context. In particular, the consideration of high switching costs (together with the consideration of low switching costs in the benefit circumstance) helps to elaborate research findings suggesting that switching costs have only a weak negative influence on relational exit and actual switching (Pick and Eisend, 2014). Specifically, the results reveal that low switching costs only work if customers consider valuable benefits from their relationships. As such, we conclude that researchers can use different switching cost levels to explain value offerings, not only an overall effect of switching costs.

### Practical Implications

This study provides a better managerial understanding of how to manage three loss categories (time, benefits, and personal loss) best in an attempt to implement an effective CRM program and reduce the probability of a customer intending to leave a relationship. Our study indicates that the upkeep category does not directly affect relationship termination; thus, less attention should be paid to this theme when designing CRM systems and more to improving time, benefit, and personal loss. However, upkeep should be considered in situations where it is known that a consumer is primarily motivated by hedonic concerns. As Figures 2C,D indicate, we also found that time and personal-loss could lead to a relationship termination for consumers with a utilitarian motivation. Because utilitarian consumers care for time and benefit in addition to personal loss, marketers need to be mindful of designing online/mobile platforms that minimize hassles (such as update requirements).

Our findings also confirm that benefit solves the CRM dilemma: that is, firms do not handle most types of CRM strategies for customer care. The findings facilitate firms’ choice and concentration in the face of CRM performance. In addition, people are likely to avoid negative emotional and financial consequences when they connect to a deep commitment with a specific object (Strachman and Gable, 2006). Thus, both shopping motivation and switching cost moderate the relationship between relationship loss and the relationship termination.

Furthermore, our findings show that relational loss categories lead to terminate a relationship when switching costs are low. However, high switching costs do not guarantee customer loyalty. Genius loyalty program is an example in the Booking.com reward program, offering big discounts and free stay based on customers’ booking records. This program is designed to stop customers from switching to competitors. However, many global travel competitors offer similar programs (e.g., silver and gold level from Expedia.com), showing that it is difficult to differentiate switching barriers. Alternatively, managers must pay particular attention to time category when time is directly involved in customer exit behavior. Therefore, online (or mobile) platforms must be configured in ways that optimize benefits and make the consumer experience time efficiency as much as possible. Conducting on-going surveys of consumers to assess their perceptions of time and modifying/improving areas perceived to be deficient could be strategies that would reduce the probability of consumers’ leaving the relationship.

Finally, most online travel firms run security programs and require unnecessary announcements requesting information updates to provide personalized experiences for their customers using Bot services. However, managing Bot services should be particularly limited in the context of upkeep loss when customers no longer want a particular relationship. For example, most online travel agencies (OTA) such as expedia.com and priceline.com use Bot services to provide optimum travel services and booking confirmation, but these services often undermine customer relationships. From an online travel firm perspective, positive effects should be maximized and negative effects should be reduced through the effective management of Bot traffics.

### Research Limitations and Further Research Directions

Although this study provides valuable contributions to theory andpractice, it has some limitations that potential future research can address. The data were collected in South Korea, a culture high on relationship orientation. In order to improve the efficacy of the findings, future research should attempt to replicate our findings from data generated in a culture very different from that of South Korea. Research shows, for instance, that the culture of the United States or many European countries would be very different from that of South Korea on Hofstede’s six dimensions (Hofstede et al., 2010). Thus, replicating our results using samples from those cultures would be a fruitful area for further research.

Similarly, another useful area for future research would be to collect consumer data during or immediately after an online visit and/or purchase. Such data would allow a deeper understanding of specific factors that can potentially affect the relationship and, as such, shed more light on our results.

In the survey carried out in this study, respondents answered questions based on their favorite travel website experiences, in which all the respondents indicated the travel websites they use most. This would be a source of heterogeneity responses if they were referring to different websites. To overcome this heterogeneity problem, we recommend exploring further studies to compare a single source (e.g., a particular website like Expedia.com) and multiple sources (e.g., hotels.com, trivago.com, lastminute.com, etc.).

Finally, we measured gender as a control variable; however, theremight be other critical variables that considered the previous levelof loyalty (Helgesen, 2006) and quality of the relationship(Storbacka et al., 1994) between the respondent and the company. This couldplay a moderating role. For example, high loyal customers mightdisplay different results from low loyal customers. On the otherhand, the relationship between low loyal customers might be lowerthan high loyal customers. Further research is required to explore these important variables when expanding the level of relationship termination.

## Data Availability Statement

The raw data supporting the conclusions of this article will be made available by the authors, without undue reservation, to any qualified researcher.

## Ethics Statement

Ethical review and approval was not required for the study on human participants in accordance with the local legislation and institutional requirements. The participants provided their written informed consent to participate in this study.

## Author Contributions

PH, and H-YH conceived the study. H-YH contributed data collection and wrote a first draft. PH analyzed the data and provided research insights.

## Conflict of Interest

The authors declare that the research was conducted in the absence of any commercial or financial relationships that could be construed as a potential conflict of interest.

## Appendix

### Measurement Scales and Factor Loadings

**Table:**

Construct/item	Loadings
Upkeep loss (α = 0.758, AVE = 0.570, CR = 0.930)	
There is the periodic requirement to change my password.	0.736
There are too many security programs.	0.754
There are unnecessary announcements requesting information updates.	0.763
I am repulsed by requiring prior consent.	0.768
Time loss (α = 0.747, AVE = 0.524, CR = 0.894)	
Joining as a member is going to be a long haul.	0.703
Finding a suitable travel product requires considerable time.	0.656
After entering a search term, most information results have no relevance.	0.762
When I shop on the travel website (or mobile application), the purchasing process is very complicated.	0.769
Benefit loss (α = 0.807, AVE = 0.533, CR = 0.973)	
The purchase requirements to achieve benefits are too high.	0.741
The benefits are insufficiently attractive to encourage me to seek a relationship.	0.742
Most benefits are unrelated to my interests.	0.699
I am uncertain how many benefits are there for spending money at the travel website.	0.708
The range of benefits is limited.	0.760
Personal loss (α = 0.831, AVE = 0.594, CR = 0.959)	
When I shop at the travel website, I have some anxiety about my personal information being exposed.	0.737
When I shop at the travel website, my big concern is the privacy issue.	0.778
The travel website makes me anxious because the purchasing process is oversimplified.	0.761
When I shop at the travel website, I have doubts about its technical stability.	0.806
Relationship termination (α = 0.817, AVE = 0.604, CR = 0.952)	
I will occasionally consider ending my relationship with the travel website.	0.721
I am actively looking for a replacement travel website.	0.821
I am unlikely to continue my relationship with the travel website.	0.786
Hedonic/utilitarian motivations (α = 0.703, AVE = 0.508, CR = 0.933)	
I accomplished only what I wanted to on this travel website visit (U).	0.714
While visiting, I just found the item(s) for which I was looking (U).	0.747
Mobile shopping satisfies my sense of curiosity (H).	0.680
I like to shop for the novelty of it (H).	0.709
Switching costs (α = 0.747, AVE = 0.606, CR = 0.905)	
It takes me a great deal of time and effort to get used to a new travel website.	0.737
It costs me too much to switch to another travel website.	0.880
In general, switching to another travel website would be a hassle.	0.711
(U) indicates the utilitarian-focused item and (H) indicates the hedonic-focused item.	
